# Retreatment of Failed Regenerative Endodontic Therapy: Outcome and Treatment Considerations

**DOI:** 10.7759/cureus.75147

**Published:** 2024-12-05

**Authors:** David Keinan, Eyal Nuni, Meital Bronstein Rainus, Tal Ben Simhon, Aaron Dakar, Iris Slutzky-Goldberg

**Affiliations:** 1 Endodontics, Tel Aviv University, Tel Aviv, ISR; 2 Endodontics, Galilee College of Dental Sciences, Nahariya, ISR; 3 Endodontics, Hebrew University Hadassah School of Dental Medicine, Jerusalem, ISR; 4 Endodontics, Rambam Medical Center, Haifa, ISR

**Keywords:** failure, guidelines, orthograde retreatment, peri-apical surgery, regenerative endodontic treatment, repeated treatment

## Abstract

Introduction

Regenerative endodontic therapy (RET) has been suggested for treating immature necrotic teeth, and failures after RET may be observed during follow-up examinations, even two years after the initial treatment. The study aimed to examine the outcomes of RET and suggest a decision-making guide for the retreatment of failed cases.

Methods

Around 414 endodontically treated immature teeth from patients aged six and 17 were screened to identify eight patients who presented with a failed RET. Data, including patients' ages, sex, medical, and dental history, were collected.

Results

The timing of failure varied significantly. Only one tooth showed failure at the three-month initial follow-up, while the remaining five teeth exhibited failures between two and seven years post-RET completion. Notably, in most instances, failure occurred after the periapical lesion had healed and root development had continued. Six teeth underwent retreatment using various approaches: repeated RET (two teeth), conventional root canal retreatment with or without an apical plug (two teeth), and surgical retreatment (two teeth). All retreatment interventions resulted in favorable outcomes.

Conclusion

Despite initial RET failure, the subsequent process of maturogenesis that followed the primary treatment led to an improved crown-to-root ratio. This resulted in a better outcome for the secondary treatment and increased patient cooperation during subsequent treatments. The study presents guidelines to aid in treatment decision-making for similar cases.

## Introduction

In recent years, regenerative endodontic therapy (RET) has been suggested for the treatment of immature necrotic teeth [1,2]. The primary aim of RET is apical healing; secondary aims include continued root development, demonstrated by both root elongation and an increase in the canal wall thickness, as well as a positive response to sensibility tests [3,4]. In a follow-up of teeth one year after RET, apical healing and complete apical closure were observed in 100% and 55% of the teeth, respectively, which were accompanied by an increase in the canal’s width and lengthening of roots [1]. Similar survival rates were reported in a recently published meta-analysis [5].

Clinically, during the final steps of RET, a layer of bio-ceramic material barrier is applied on the scaffold within the root canal space [3,4]. There is limited evidence as to the effect of the scaffold, either platelet-rich plasma (PRP), platelet-rich fibrin (PRF), platelet pellet, or blood clot, on the secondary outcomes, although all treatment modalities resulted in healing of the apical rarefaction [4]. A retrospective study reported lower success and survival rates after RET in traumatized immature necrotic teeth. The type of traumatic injury had an effect on the treatment outcome, with teeth after avulsion having the lowest survival and success rates (66.7% and 33.3%, respectively) [6]. The etiology of pulp necrosis, which led to treatment, either trauma, dens evaginatus, or caries, did not affect the success of the treatment, which ranged between 93.1% to 96% [7]. Histological examinations of extracted human teeth that have undergone regenerative endodontic therapy (RET) have shown that the newly formed tissues primarily consist of fibrous connective tissue and cementum-like connective tissue [8,9]. 

Failures after RET may be observed during follow-up examination. In a systematic review of failed RET cases, 39% of the 67 failures were detected more than two years after initiation of the RET [10]. According to a study that analyzed 16 failed cases, 37.5% of the failures were identified within six months after the procedure. The main etiologic factors that can lead to failure are dental trauma, dens evaginatus, and dental caries [11]. Interventions after failures include extraction, nonsurgical root canal treatment, repeated RET, and Ca(OH)₂ apexification [10-12], as well as a surgical approach [11]. There is scarce clinical evidence regarding the outcome of treatment of failed RET cases [12-14]. In a report of three failed root canal treatment (RET) cases, the teeth were retreated after repeated debridement of the root canals. All the cases were treated in a single visit using negative-pressure irrigation. Orthograde root canal treatment was performed in one case, and in a second case, orthograde retreatment was supplemented by an apical mineral trioxide aggregate (MTA) prior to obturation. In the third case, repeated RET was performed. In all three cases, resolution of the periapical lesion was observed [12]. Successful repeated RET was observed in another case report describing the failure of a previously healed immature incisor tooth. The tooth was retreated in one visit and showed complete apical repair after two years [14].

The main reason for failure is a persistent bacterial infection [10]. A more significant decrease in primary microflora was found in successful RET cases than in failed cases treated with either Ca(OH)₂ or chlorhexidine. After dressing with Ca(OH)₂ or chlorhexidine, a greater reduction in microflora was observed in successful RET cases compared to failed treatments [15]. It is assumed that lack of mechanical debridement during the regenerative procedure allows for relapse of the apical pathology after a temporary resolution of the signs and symptoms [12]. This study aims to describe the outcome of retreatment in failed cases of regenerative treatment, describe the reasons for the failure, discuss the optional treatment options, and present a decision-making flowchart.

## Materials and methods

The study was conducted in the Hebrew University Hadassah School of Dental Medicine in Jerusalem. Ethical approval was obtained from the Institutional Helsinki Ethics Committee (HMO-21-0231). Data collection was based on the digital medical records of patients treated by endodontic post-grad students between 2015 and 2020. From patients aged six to 17, 414 endodontically treated immature teeth (Nolla stages 7-9) [15] were screened to allocate patients with a failed RET. Data including patients' ages, sex, medical, and dental history were collected. Their dental records, specifically regarding the treated tooth, were compiled. Cases with incomplete records or insufficient follow-up were excluded. Around 48 teeth underwent RET and were followed for one year or more. Eight failed cases were observed among the teeth that underwent RET. Two teeth were referred for extraction due to a root fracture. Six patients (aged seven to 16 years) who were referred for the management of apical periodontitis that developed after a previous RET failed were retreated and followed for more than one year. The teeth included five maxillary incisor teeth and one maxillary second premolar (Table 1).

**Table 1:** Initial regenerative endodontic treatment (demographics, etiology, treatment details, and outcomes) PN: pulp necrosis; ASAP: asymptomatic apical periodontitis; SAP: symptomatic apical periodontitis; AAA: acute apical abscess; MTA: mineral trioxide aggregate; BC putty: EndoSequence® RRM; PCO: pulp canal obliteration

Case	Age (y) -RET	Sex	Tooth	Etiology		Nolla	Diagnosis	Intracanal barrier	Outcome
1	7	F	21	Trauma	Extrusion	7	PN, ASAP	MTA	Maturogenesis & PCO
2	8	M	11	Trauma	Lateral luxation	8	PN, ASAP	MTA	Intracanal calcified bridge
3	13.5	M	11	Trauma	No data	8	PN, SAP	MTA	Intracanal calcified bridge
4	13	M	15	Tooth morphology	Dens invaginatus	8	PN, ASAP	MTA	Maturogenesis & PCO
5	8	M	11	Trauma	Crown fracture	8	PN, SAP	MTA	Apical repair
6	9	M	11	Trauma	Extrusion	7	PN, AAA	EndoSequence® RRM	Complete periradicular repair

All patients underwent clinical and radiographic examinations. In cases where apical surgery was deemed necessary, CBCT scans were also conducted. After evaluating various treatment options, the teeth were successfully treated (Table 2).

**Table 2:** Retreatment of failed regenerative endodontic treatment cases RET: regenerative endodontic therapy; PT: previously treated; ASAP: asymptomatic apical periodontitis; SAP: symptomatic apical periodontitis; Nolla stage 7: third of the root completed; Nolla stage 8: two thirds of the root completed; Nolla stage 9: root almost completed, but the apical foramen is still open * Data includes the timing of failure diagnosis, mode of treatment, follow-up, and outcome

Case	Timing of failure	Diagnosis of failure (in years, post RET)	Nolla	Diagnosis	Etiology	Intervention	Follow-up (in years)	Outcome
1	Late	6	10	PT, ASAP	Unknown	RCT	1	Healed
2	Late	7	8	PT, SAP	MTA disintegration (following orthodontics)	RCT with apical plug	1	Healed
3	Late	2.5	8	PT, ASAP	Coronal leakage	Surgical	2	Complete healing
4	Late	2	10	PT, SAP	Untreated canal	Surgical	3	Incomplete healing
5	Late	3	8	PT, SAP	Persistent infection	Re- RET	2	Healed
6	Immediate	0.25	7	PT, ASAP	Secondary trauma	Re-RET	1	Healed

## Results

Reviewing the medical records of the young patients treated in the endodontic post-grad clinic revealed 48 well-documented cases of RET, eight of which failed. Initially, 46 of the cases were successful, with one immediate failure recorded and one tooth extracted because of infection after one year. This resulted in an initial survival rate of 97.9% (47 out of 48 teeth) and an initial success rate of 95.8% (46 out of 48 teeth). Over time, one additional tooth was extracted due to root fracture, leading to a long-term survival rate of 95.8% (46 out of 48 teeth) and a long-term success rate of 83.3% (40 out of 48 teeth).

Case 1

A seven-year-old female patient who was diagnosed with pulp necrosis and asymptomatic apical periodontitis of the left maxillary central incisor following a complicated crown fracture and traumatic extrusion was treated by RET according to the American Association of Endodontists (AAE) guidelines. The immature tooth was classified as Nolla stage 7, and the plug placed in the canal during the second visit was MTA ProRoot (Dentsply Sirona, Johnson City, TN). The girl was scheduled for follow-up appointments, and after 1.5 years, continued root development, and the periapical lesion has healed.

**Figure 1:**
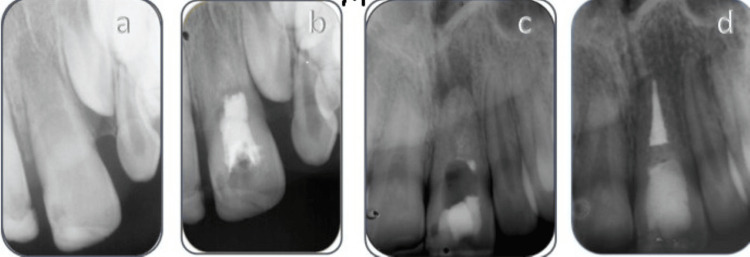
Orthograde retreatment of a failed regenerative endodontic treatment a: preoperative radiograph; b: postoperative radiograph following RET, the intracanal barrier was MTA; c: six years after RET, the root completed its development, and complete obliteration of the root canal can be observed (the referring dentist removed the MTA plug after a larger periapical radiolucency was observed in the radiograph); d: a one-year follow-up radiograph demonstrating the complete healing of the periapical lesion. MTA: mineral trioxide aggregate; RET: regenerative endodontic therapy

Six years later, a periapical lesion reemerged, and her dentist referred her after unsuccessfully attempting to perform a root canal treatment. The root was fully developed and was now classified as Nolla 10, and pulp canal obliteration (PCO) was observed. During the orthograde retreatment, the root canal was negotiated using ReadySteel C+ files (Dentsply Maillefer, Switzerland) under the operative microscope. After ultrasonic irrigation, the canal was obturated with gutta-percha and AH Plus root canal sealer (Dentsply, DeTrey, Konstanz, Germany), and the access cavity was sealed with a composite restoration. After a one-year follow-up, the periapical lesion healed (Figure 1).

Case 2

An eight-year-old male patient who was diagnosed with asymptomatic apical periodontitis for the right maxillary central incisor following lateral luxation was treated by RET according to the AAE guidelines. The immature tooth was classified as Nolla state 8, and the plug placed in the canal during the second visit was MTA ProRoot (Dentsply Sirona Johnson City, TN). The boy was scheduled for follow-up appointments, and after three years, the periapical lesion had healed, and a calcified barrier was observed apical to the MTA plug. The root canal remained without any additional change.

**Figure 2:**
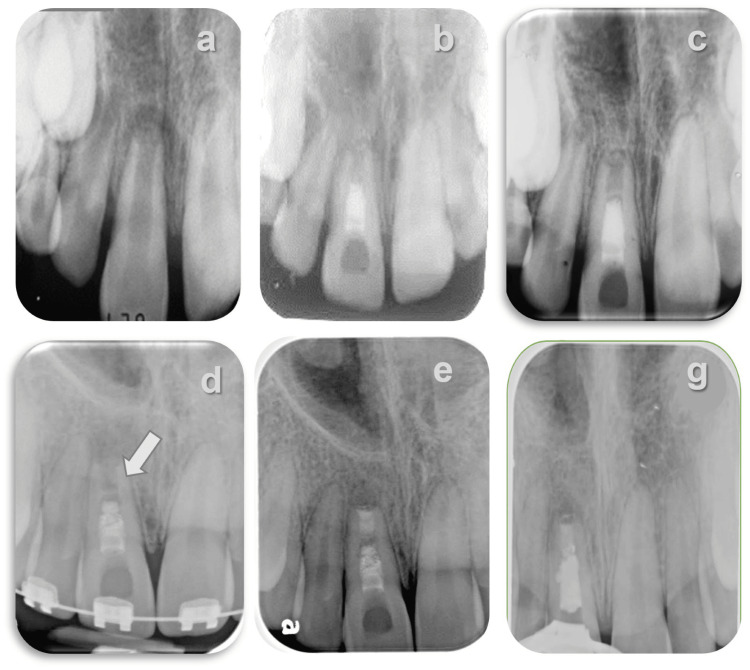
Orthograde retreatment and apical plug placement following disintegration of the MTA plug a: preoperative radiograph showing an immature tooth with a periapical lesion; b: postoperative radiograph following RET, an MTA intracanal barrier was placed; c: complete healing is observed three years after RET, although the apical foramen remained open and an intra-canal calcified bridge can be seen apical to the MTA plug; d: seven years after RET, during orthodontic treatment, a disintegration of the MTA plug, marked by an arrow, was evident and a radiopaque calcified bridge had formed apical to the MTA plug; e: following biomechanical preparation, an apical bioceramic plug using EndoSequence® RRM was placed and radiopaque remnants of the MTA are still visible on the canal walls; g: one year after completing the retreatment, radiographic evidence showed healing of the periapical lesion. MTA: mineral trioxide aggregate; RET: regenerative endodontic therapy

Seven years after the RET, he was referred for treatment by his orthodontist due to a reemerging periapical lesion. The MTA plug appeared to be disintegrated. After the composite restoration of the access cavity was removed, the remnants of the MTA plus were removed. Following chemomechanical preparation, and since the apical foramen diameter was very wide, an apical plug was placed using EndoSequence® RRM (Brasseler, USA). After a one-year follow-up, the periapical lesion healed (Figure 2).

Case 3

A 16-year-old male patient was diagnosed with pulp necrosis and symptomatic apical periodontitis for the right maxillary central incisor following a traumatic dental injury that occurred two and a half years earlier. The exact nature of the dental injury was unknown. According to his dental records, the boy was treated by RET following the AAE guidelines. The immature tooth was classified as Nolla state 8, and the plug placed in the canal during the second visit was MTA ProRoot (Dentsply Sirona, Johnson City, TN).

**Figure 3:**
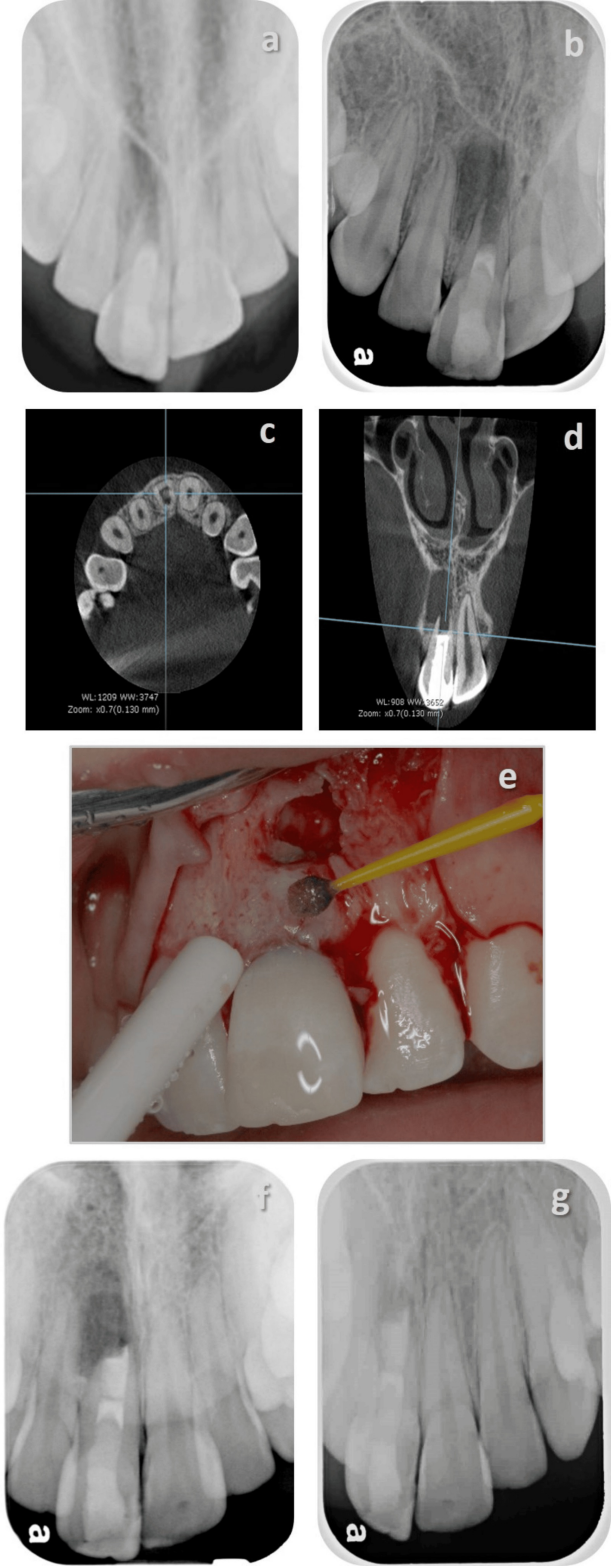
Surgical retreatment following a failed regenerative endodontic treatment a: one-year follow-up after the initial regenerative endodontic therapy, the intra-canal barrier was MTA; b: 2.5-year follow-up demonstrating a large periapical radiolucent lesion and a calcified bridge can be observed apical to the MTA plug; c: CBCT axial view demonstrating the incomplete calcified bridge under the MTA plug; d: CBCT coronal view demonstrating the thin radicular walls, the immature apex, and the large hypodense lesion; e: following root-end resection, a micro brush soaked in chlorhexidine 2% was used to scrub the canal walls, and a root-end filling was placed using EndoSequence® RRM; f: immediate post-op; g: a two-year follow-up demonstrating incomplete healing. MTA: mineral trioxide aggregate; RET: regenerative endodontic therapy; CBCT: cone-beam computed tomography

The boy returned 2.5 years after completion of the RET due to the re-emergence of the periapical lesion. Considering the large MTA plug, the calcified barrier apical to the plug, and the thin dentinal walls, apical surgery was considered. The patient was scheduled for apical surgery. During the procedure, the canal was only minimally resected, and the canal walls were irrigated and scrubbed with a micro-brush with chlorhexidine 2%. The empty root canal space was filled with EndoSequence® RRM. At the two-year follow-up, incomplete healing was observed (Figure 3) [16].

Case 4

A 15-year-old male patient who was diagnosed with asymptomatic apical periodontitis of the right maxillary second premolar was referred by his orthodontist before the beginning of an orthodontic treatment. RET was performed two years earlier, following the AAE guidelines using MTA, because of pulp necrosis that developed because of the irregular type II dens invaginatus anatomy.

**Figure 4:**
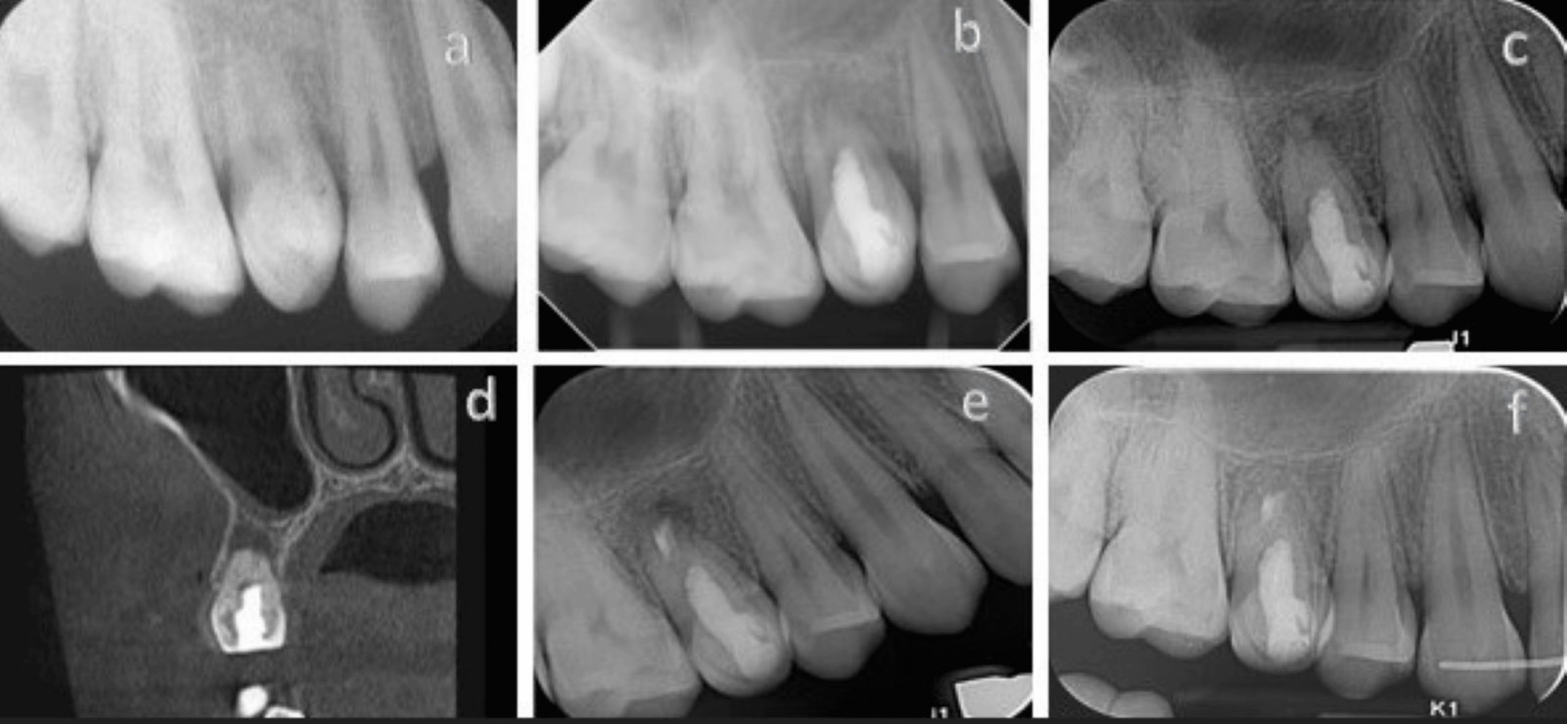
Retrograde retreatment of a failed regenerative endodontic case a: a maxillary second molar with dens invaginatus Class I diagnosed with pulp necrosis and an immature root, Nolla stage 8; b: RET was performed using MTA as the intracanal barrier; c: two years after the RET, continued root development and pulp canal obliteration were observed, an apical rarefaction is demonstrated; d: CBCT scan, coronal view of the tooth demonstrates the apical radiolucency and the obliteration of the canal space; e: post-operative radiograph of the tooth after apicoectomy, the root-end filling is EndoSequence® RRM; f: complete apical repair can be observed three years after the apical surgery and orthodontic treatment. RET: regenerative endodontic therapy; CBCT: cone-beam computed tomography; MTA: mineral trioxide aggregate

Upon arrival, it was evident that the root continued to develop, although a periapical lesion was now evident. A CBCT scan revealed that the dens had been only partially sealed, and the canal apical to the intra-coronal plug was obliterated. Following the replacement of the coronal seal, apical surgery was performed. The short root canal was only minimally resected. Debridement of the apical root canal was done using ultrasonic tips, and retrograde obturation was done using EndoSequence® RRM. After one year, complete healing of the apical radiolucency was observed, and orthodontic treatment was initiated. Three years after the completion of the RET, the periapical tissue remained normal (Figure 4).

Case 5

A 10-year-old male patient who suffered a crown fracture and pulp necrosis with asymptomatic apical periodontitis in the right maxillary central incisor was treated by RET using MTA following the AAE guidelines. Apical repair was observed during the follow-up examination, and the tooth remained asymptomatic. 

**Figure 5:**
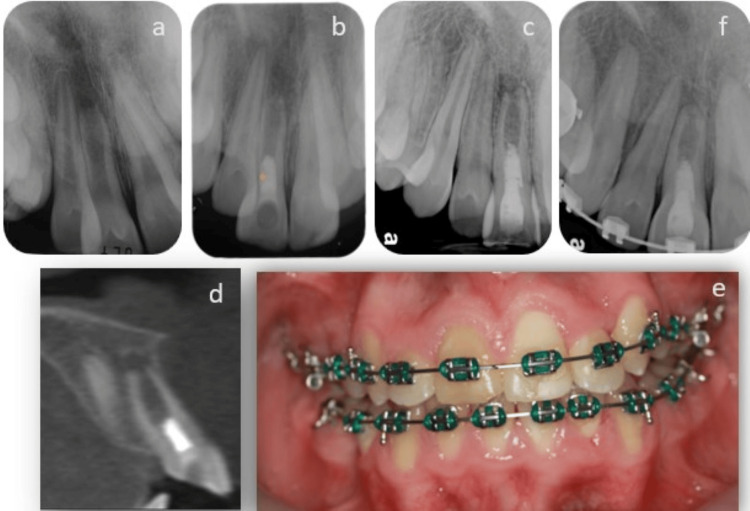
Repeated regenerative endodontic treatment after failue of the initial treatment a: preoperative radiograph, a large periapical radiolucency can be observed; b: postoperative radiograph following RET; c: incomplete healing of the periapical lesion can be observed two years after completion of the RET; d: a CBCT scan demonstrated the blunt immature apex and a small periapical lesion; e: discoloration of the treated tooth, which was orthodontically treated, two years after completion of the RET; f: two years after ReRET, complete healing of the periapical lesion can be observed, and an apical barrier is demonstrated, and an increase in the thickness of the walls can be seen. RET: regenerative endodontic therapy; CBCT: cone-beam computed tomography

Two years later, his orthodontist referred the patient due to an acutization of an apical radiolucency. A periapical radiograph and a CBCT scan demonstrated an increase in the thickness of the root canal walls, with the root end becoming blunt and almost closed. Repeated RET resulted in complete repair of the periapical lesion and a calcified apical tissue, observed two years later. After apical healing, the orthodontic treatment was continued (Figure 5).

Case 6

A nine-year-old boy presented with a horizontal root fracture in the right central maxillary incisor following the tooth's extrusion. A periapical radiograph revealed a type II dens invaginatus. The tooth was diagnosed with pulp necrosis, and inflammatory root resorption was observed. RET was performed according to the AAE guidelines using calcium hydroxide dressing between visits. After bleeding was allowed into the canal space, a collagen plug was placed as a barrier covered by EndoSequence® RRM. Eight weeks after its initiation, treatment was completed, and healing of the radiolucent lesion was evident.

**Figure 6:**
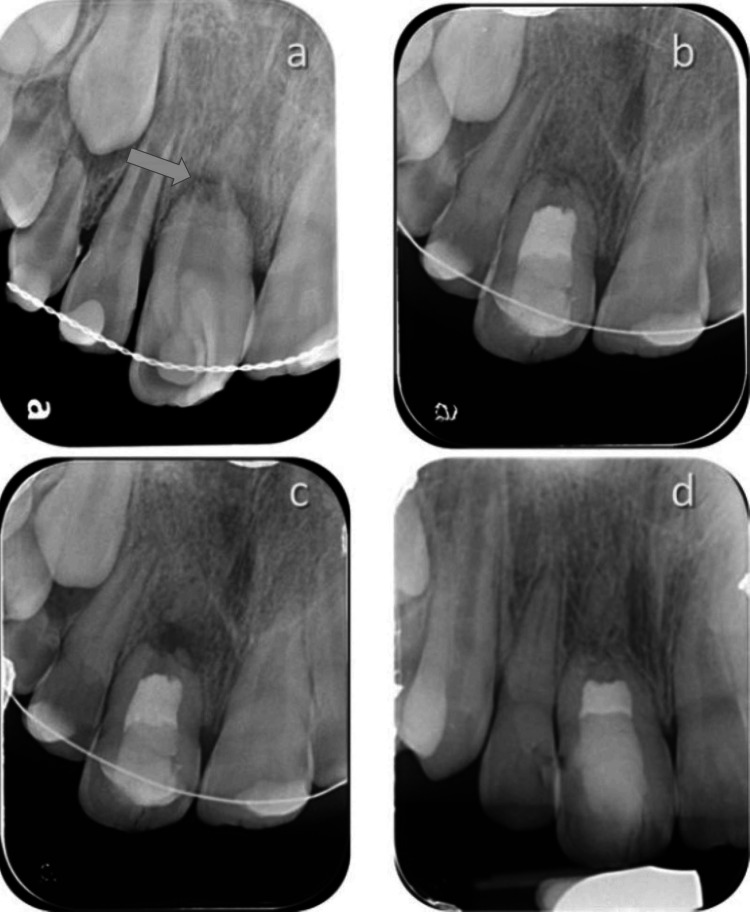
Repeated regenerative endodontic treatment after failure of the initial treatment a: preoperative radiograph shows a Class I dens invaginatus, a horizontal root fracture is observed, separating the coronal and apical segments (marked by an arrow); b: an immediate postoperative radiograph taken after completion of regenerative endodontic treatment shows that the radiolucent lesion at the fracture line has healed completely; c: a follow-up radiograph reveals a new radiolucent lesion between the coronal and apical segments three months after the procedure; d: a one-year follow-up after repeated regenerative treatment shows complete healing of the lesion, with bone-like tissue filling the root canal space in the coronal segment and pulp canal obliteration in the apical segment, continuous periodontal ligament surrounds both segments (Courtesy of Z Margy and I Slutzky-Goldberg).

During follow-up, the tooth exhibited recurrent pathology at the fracture line. The patient was scheduled for retreatment of the tooth. Repeated RET included additional ultrasonic irrigation and calcium hydroxide dressing. One year after retreatment, the apical rarefaction resolved with continued maturogenesis of the apical fragment and approximation of the coronal and apical fragments attached by a calcified tissue and surrounded by the continuous periodontal ligament (Figure 6).

## Discussion

Regenerative endodontics has become a promising alternative for treating immature teeth with apical pathology [3]. A recent meta-analysis reported a 100% survival rate for immature teeth after RET for at least one year [5]. Similar success rates that ranged between 93.1% and 96% were found for teeth that underwent RET due to trauma, dens evaginatus, or caries [7]. These results are similar to the one-year survival and success rates reported in the current study (97.9% and 95.8%, respectively). Despite high survival and success rates [3-5], failures can still occur, presenting clinicians with challenging treatment decisions, particularly in immature teeth [11,12,17]. Despite initial signs of healing and continued root development observed during short-term follow-up examinations, six cases ultimately failed. These cases were successfully managed through secondary interventions.

The timeframe during which RET failures are detected varies significantly, ranging from less than six months following treatment [11] to as long as four, eight, and even 11 years [12]. In the literature, the average follow-up time reported was shorter: 19.9 months for the mineral trioxide aggregate plug and 16.7 months for the RET group [18].

In the current study, one tooth was extracted immediately after the RET due to persistent infection, and one failure was observed three months after the initial follow-up visit, while the other five cases showed failures between two and seven years after the RET was completed, and another tooth was extracted due to a root fracture, despite initial signs of success. This study emphasizes that while short-term success is encouraging, it does not guarantee long-term stability in RET cases. The success rate dropped over time from 95.8% to 83.3%. The study strongly advocates for extended, diligent monitoring to ensure the best possible outcomes for patients undergoing regenerative endodontic procedures.

The initial success of the regenerative treatment was observed during the follow-up in the cases described above. It was manifested in the healing of the periapical lesion and the continued development of the roots. However, over time, a new periradicular rarefaction reemerged. RET failures have been attributed to persistent infection [10] or inadequate root canal disinfection due to a lack of mechanical debridement, which is associated with an effort to avoid further weakening of the thin and fragile dentinal walls [19]. Indeed, histological examination after RET’s failure demonstrated bacteria and biofilm on the canal walls [9].

It was reported that most of these failures were following a secondary dental trauma (56%), as observed in case number 6; abnormal anatomy of the dens invaginatus (25%), as observed in case number 3; and a result of persistent infection, as observed in case 5 [11]. Failures that occur after the initiation of orthodontic treatment [17], as reported in case number 2, raise concerns as to the immune competency of the revitalized tissue after regenerative endodontic procedure (REP) [17]. Teeth treated by RET may not withstand biting forces and fracture, especially if they are poorly developed and the canal walls are short and thin [19]. Thus, they necessitate extraction, as they may not be restorable [20].

**Figure 7:**
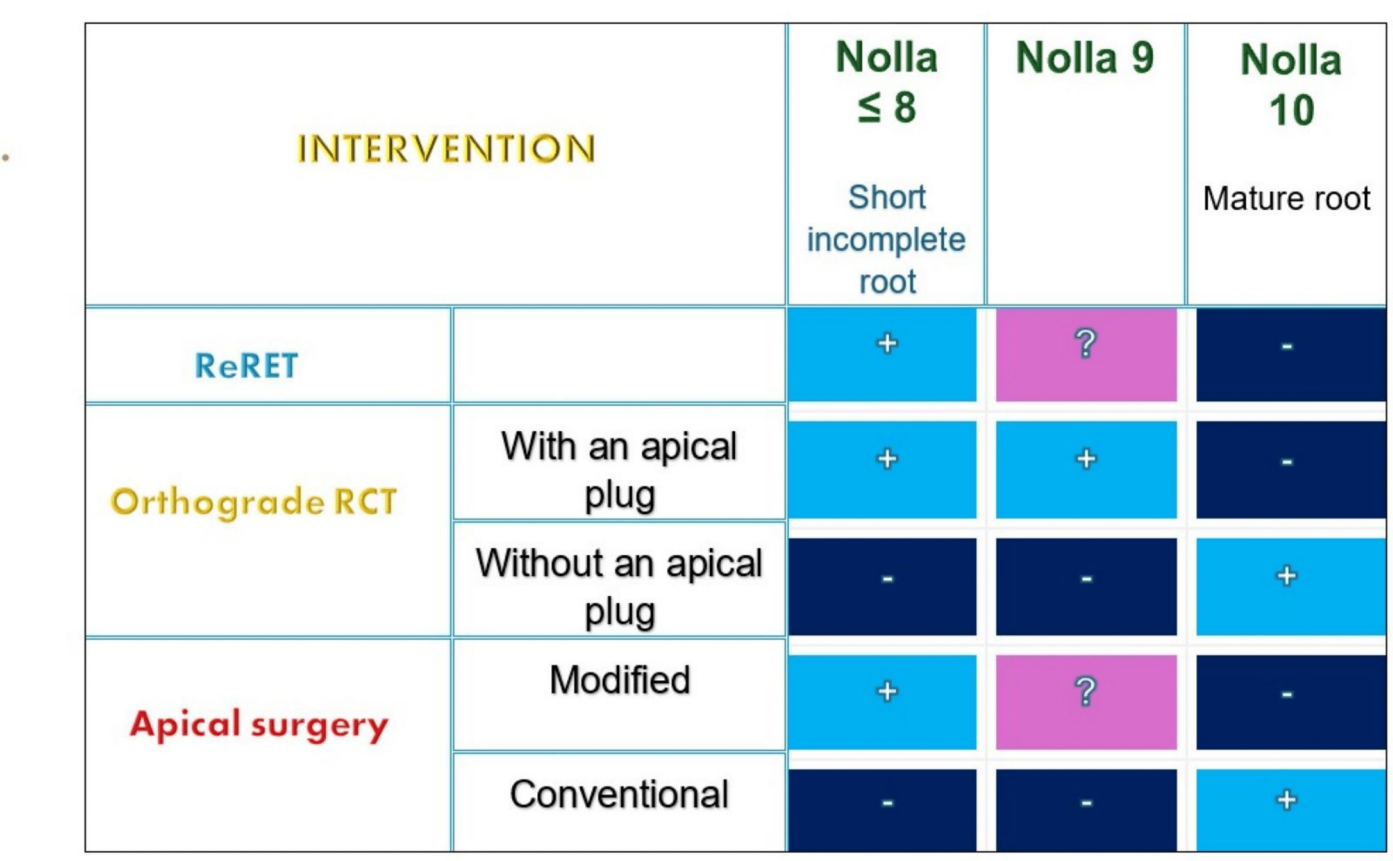
Suggested interventions for regenerative endodontic treatment failure Type of intervention according to the degree of root development: light blue (+): this type of intervention is recommended; dark blue (-): this type of intervention is not recommended; pink (?): this type of intervention can be considered if the root is short, but the apical foramen is almost closed. RCT: root canal treatment

Regenerative treatment failures can be successfully treated by several treatment options, including repeated RET [12], orthograde root canal treatment with or without creating an apical barrier with a bio-ceramic material, or performing a surgical intervention (Figure 7).

Repeated RET should be considered in an attempt to improve the low prognosis resulting from the thin root walls and the unfavorable crown-to-root ratio. RET is likely to fail without adequate root canal disinfection [21]. Efforts are being made to improve the efficacy of the antibiotic paste placed in the canals during RET [22,23]. Current disinfection protocols applied during RET cannot eliminate bacterial infection from the root canals [24]. Supplementary agitation of sodium hypochlorite by means such as ultrasonic activation or the use of XP finisher (FKG Dentaire SA, La Chaux-de-Fonds, Switzerland) can effectively reduce the biofilm and enhance disinfection of the root canal [23,24], especially in wide root canals [25].

In the event of a failed regenerative endodontic procedure (REP), the procedure can be repeated, offering another opportunity for success in cases where the initial REP did not yield the desired outcomes [10,12,14]. Modifying the protocol during repeated procedures and treatments may be necessary, allowing for variations in the procedure. The current protocol includes the use of several antimicrobial agents [3]. Alternatives like calcium hydroxide or modified TAP can be considered, along with a different intra-coronal barrier. Another bioceramic material, such as Biodentine, which has different characteristics and setting times, may perform better [26].

RET was successfully repeated in two cases. The successful treatment outcome in case number 6 after repeated RET can be attributed both to the patient's young age and the wide diameter of the canal at the fracture line [27]. This may imply that when a failure occurs in teeth with a large apical diameter, especially when the patient is young, the preferred treatment modality will be a repetition of the RET. However, a surgical approach may be required when a calcified barrier has formed apical to the bioceramic plug in the canal (Figure 2).

Surgical endodontics can be a viable option to address regenerative endodontic failures. The main advantage of this procedure in teeth with short roots is maintaining the crown-to-root ratio. It is usually recommended to resect 3 mm of the root end during apical surgery to remove the majority of ramifications and lateral canals [28]. Since the apical canal is quite wide, it may be advisable to minimally resect the root by one mm or to smooth out any uneven edges of the root.

Favorable outcomes were reported after minimal root resection and extended retrograde root end preparation [29]. This approach can also be suggested when the orthograde access to the infected tissue in the canal is blocked. Removal of the bioceramic plug from the canal may risk the tooth in fracture. When anatomical constraints prevent apical surgery, intentional replantation (IR) can be an alternative treatment option. A case report highlights the successful intentional replantation of an immature central incisor that had undergone RET and later developed a chronic apical abscess. Despite signs of external root resorption, the replanted tooth remained symptom-free throughout a four-year follow-up period [30].

In most cases, failure occurred after the healing of the periapical lesion and continued root development. Despite the late failure, it can be assumed that teeth with a better crown-to-root ratio [31] and the increase in the root wall thickness are advantageous compared to more immature teeth, which are more susceptible to fracture [19]. Additionally, the time between regenerative endodontic therapy (RET) and the decision to perform a full root canal treatment (RCT) due to reemerging pathology may allow for greater patient cooperation, particularly among young individuals.

## Conclusions

Teeth that have undergone successful RET and demonstrated treatment failure at the follow-up appointments can undergo additional treatment that will allow the preservation of the teeth and healing of the periapical lesion. The treatment options include ReRET, orthograde root canal treatment with or without an apical plug, or surgical root canal treatment. Despite the initial treatment failure, a successful outcome was observed after a secondary intervention.
